# Cognitive framing modulates emotional processing through dorsolateral prefrontal cortex and ventrolateral prefrontal cortex networks: A functional magnetic resonance imaging study

**DOI:** 10.1002/brb3.1761

**Published:** 2020-08-04

**Authors:** Ulrich Kirk, Lau Lilleholt, David Freedberg

**Affiliations:** ^1^ Department of Psychology University of Southern Denmark Odense Denmark; ^2^ Department of Psychology University of Copenhagen Copenhagen Denmark; ^3^ Department of Art History and Archaeology Columbia University New York NY USA; ^4^ Italian Academy for Advanced Studies Columbia University New York NY USA

**Keywords:** amygdala, dorsolateral prefrontal cortex, emotion, framing, functional magnetic resonance imaging, ventrolateral prefrontal cortex

## Abstract

**Introduction:**

In this study, we show new evidence for the role of ventrolateral prefrontal cortex‐dorsolateral prefrontal cortex (VLPFC‐DLPFC) networks in the cognitive framing of emotional processing.

**Method:**

We displayed neutral and aversive images described as having been sourced from artistic material to one cohort of subjects (i.e., the art‐frame group; *n* = 19), while identical images, this time identified as having been sourced from documentary material (i.e., the doc‐frame group; *n* = 20) were shown to a separate cohort.

**Results:**

Using functional magnetic resonance imaging (fMRI), we employed a linear parametric model showing that relative to the doc‐frame group the art‐frame group exhibited a modulation of amygdala activity in response to aversive images. The attenuated amygdala activity in the art‐frame group supported our hypothesis that reduced amygdala activity was driven by top‐down DLPFC inhibition of limbic responses. A psychophysiological interaction (PPI) analysis demonstrated that VLPFC activity correlated with amygdala activity in the art‐frame group, but not in the doc‐frame group for the contrast [Aversive > Neutral].

**Conclusion:**

The role of the VLPFC in cognitive control suggests the hypothesis that it alongside DLPFC insulates against embodied emotional responses by inhibiting automatic affective responses.

## Graphical abstract

We show that cognitive framing have neural effects on emotional processing.

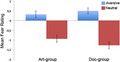

## 1 INTRODUCTION

No single aspect of our mental life is more profound than our ability to experience emotions. Emotions are what makes our lives interesting, gratifying, and worth living. For decades' researchers have sought to understand how we process emotional stimuli and regulate emotional experiences, an endeavor that has provided us with numerous valuable insights. Furthermore, studies have investigated how contextual factors influences the way in which emotional stimuli are processed (Aldao, 2013; Aldao & Tull, 2015; Gross, 2015).

In one of the studies examining this question, Gerger, Leder, and Kremer (2014) illustrated that framing emotional stimuli as art profoundly alters people's emotional experience. More specifically, the results from this study indicated that framing highly aversive pictures as art decreases the intensity of emotional experiences and allow people to appraise aversive stimuli more positively. Although Gerger et al. (2014) provide evidence that framing aversive stimuli as art alters the way in which people appraise and experience aversive stimuli, very little is known about the neurobiological underpinnings responsible for this apparent framing effect. The purpose of the present study is therefore to investigate how highly aversive pictures framed as art are processed compared to identical pictures framed as documentary photographs by using functional magnetic resonance imaging (fMRI).

It is assumed that framing stimuli as art provides the cognitive system with cues on how to process and respond to objects with artistic qualities^4^. For instance, Leder and colleague (Leder, Belke, Oeberst, & Augustin, 2004) argue that framing stimuli as art give rise to certain expectations such as the belief that one will have a gratifying and positive emotional experience. Furthermore, scholars have argued that framing stimuli as art enables a specific style of perception which allow people to adopt a more distanced perspective on what is depicted and appraise the aesthetic qualities of the artwork (Cupchik, 2002). In support of this view, multiple studies have shown that people engage in more elaborative processing (Nadal, Munar, Capo, Rossello, & Cela‐Conde, 2008) and attend more to stylistic and formal properties of visual stimuli, when percepts are placed in an art context (Cupchik, Vartanian, Crawley, & Mikulis, 2009; Jacobsen, Schubotz, Höfel, & Cramon, 2006; Kirk, Skov, Hulme, Christensen, & Zeki, 2009). Lastly, placing visual stimuli in an art context signals that the visual content is just a figment of the artist's imagination and can be considered as fictional (Gerger et al., 2014).

Previous research indicates that appraising an aversive stimulus as fictional rather than realistic profoundly reduces its emotional impact (Mocaiber et al., 2010, 2011). Furthermore, it has been shown that adopting a psychological distance from aversive stimuli reduces signs of arousal and bodily emotional expressions (Gross, 1998; Lazarus & Alfert, 1964; Speisman, Lazarus, Mordkoff, & Davison, 1964). Hence, evidence suggest that placing stimuli in an artistic context fosters appraisal processes that allows the observer to suppress his or her initial emotional reaction and judge aversive stimuli more positively (Gerger et al., 2014). Consequently, it seems plausible that framing aversive stimuli as art leads to increased neural activity in brain regions associated with top‐down appraisal processes, thereby allowing the observer to perceive aversive stimuli differently and have a positive emotional experience.

Neuroimaging studies have shown that several brain regions play an important role in top‐down appraisal processes, such as cognitive reappraisal and attentional control. For instance, Ochsner, Bunge, Gross, and Gabrieli (2002) found that the neurobiological underpinnings of cognitive reappraisal are associated with increased activation in the lateral and medial prefrontal regions and decreased activation in the amygdala and medial orbitofrontal cortex. Similarly, both Urry et al. (2006) and Delgado, Nearing, LeDoux, and Phelps (2008) showed that cognitive reappraisal influences amygdala activity through connections to regions of the ventromedial PFC. Furthermore, Kanske, Heissler, Schönfelder, Bongers, and Wessa (2011) found that both cognitive reappraisal and distraction strategies rely on control areas in the medial and dorsolateral prefrontal and inferior parietal cortex, and leads to decreased activity in the bilateral amygdala.

In almost all studies on emotion processing and regulation, subjects are either specifically instructed to employ a reappraisal strategy or utilize attentional control in order to effectively alter the emotional impact of a stimulus, or to use a “natural” mode of perceiving the emotionality of the stimulus, without any specific instruction. As opposed to this, the present study extends this line of research by investigating whether aversive pictures framed as art promote elevated levels of neural activity in regions responsible for top‐down appraisal processes. Using aversive and neutral images taken from IAPS database (Lang, Bradley, & Cuthbert, 1997), we told one group of subjects that they were viewing images displayed in museums of contemporary art, while the other group was told that the images were sourced from documentary material and thus depicted real‐life events. Thus, in order to probe the cognitive modulation of the emotional experience, we changed the cognitive frame in which the same highly arousing stimuli were presented.

Following the literature on emotion processing and regulation (Delgado et al., 2008; Kanske et al., 2011; Ochsner et al., 2002; Urry et al., 2006), we hypothesized that amygdala activity in responding to aversive images would be attenuated in the case of subjects belonging to the art‐frame group, while in the case of the doc‐frame group such activity would be comparatively elevated. Furthermore, in line with previous research (Delgado et al., 2008; Kanske et al., 2011; Ochsner et al., 2002; Urry et al., 2006), we hypothesized that the emotional response of subjects in the art‐frame group would be modulated by increased activity in regions associated with top‐down appraisal processes, such as dorsolateral prefrontal cortex (DLPFC). Finally, given that DLPFC and ventrolateral prefrontal cortex (VLPFC) have been shown to be interconnected both in nonhuman primates (Barbas, 2000; Barbas & Pandya, 1989; Yeterian, Pandya, Tomaiuolo, & Petrides, 2012) and humans (Goulas, Uylings, & Stiers, 2012), we further hypothesized that VLPFC alongside DLPFC would be associated with decreased amygdala activity in the art but not the doc‐frame group.

## 2 MATERIAL AND METHODS

### 2.1 Subjects

To test our hypotheses, we enrolled thirty‐nine subjects in an fMRI paradigm and divided them into two groups: an art‐frame group and a doc‐frame group. The art‐frame group consisted of 19 subjects and the doc‐frame group of 20 subjects. The art‐frame group included eight women and 11 men (mean age 22.7), while the doc‐frame group included nine women and 11 men (mean age 24.2). The two groups did not differ in terms of mean age or gender distribution and were randomly assigned to either the art or the doc‐frame group. None of the subjects were educated in the arts. All subjects had normal or corrected‐to‐normal vision, and none had a history of neurological or psychiatric disorders. All procedures and experiments reported involving human subjects were approved and conducted in accordance with the Institutional Review Board of Virginia Tech. All volunteers in the study participated in the experimental tasks after giving informed consent.

### 2.2 fMRI task

#### 2.2.1 Image‐viewing paradigm

All subjects were scanned in a passive task involving viewing neutral and aversive IAPS images. Prior to scanning, subjects in the two groups were given a different set of instructions. The art‐frame group received the following instructions: “Today you will be viewing images of artwork while you undergo an MRI scan. Some of these images have a very strong emotional effect. Inside the scanner you will be presented with 80 photographs that have been exhibited in contemporary art museums as works of art.” Subjects in the doc‐frame group received the following instructions: “Today you will be viewing photographs while you undergo an MRI scan. Some of these images have a very strong emotional effect. Inside the scanner you will be presented with 80 photographs that depict real‐life events.”

In the scanner, subjects were presented with 80 IAPS images selected from the IAPS database (Lang et al., 1997). Specifically, subjects were presented with images belonging to two emotional categories, namely 40 aversive images (valence: mean 2.9, range 1.7–3.9; arousal mean 5.5, range 3.5–7.4) and 40 neutral images (valence: mean 5.1, range 4.23–5.9; arousal mean 3.1, range 1.7–5.2). The procedure was presented in a pseudorandomized fashion, and picture order was counterbalanced across subjects. During the scanning session, subjects were instructed to passively view the images. Postscanning, subjects were asked to complete an unexpected task of behavioral rating of the images, while making a self‐paced subjective aversion rating using a Likert scale (1–7). It is important to emphasize that subjects were not instructed about this subsequent behavioral rating‐task prior to the scanning session. The same images were presented to both groups with the only difference being the initial framing of the images presented as either art or documentary. In the behavioral task, the images were displayed in a randomized order compared with the scanning session. Prior to the behavioral task, subjects were specifically instructed to rate how frightening each images were perceived in the moment on a scale ranging from 1 to 7 (1 = not frightening; 7 = extremely frightening). We used an event‐related fMRI design. On each trial, an image was presented for 5 s, followed by an inter‐trial interval for 4–14 s. The images were presented at a screen resolution of 1,024 × 768 pixels and centered in a 500 × 500‐pixel resolution surrounded by a black background. Stimuli were presented, and responses collected using NEMO (Human Neuroimaging Lab, Virginia Tech Carilion Research Institute). The stimuli were back‐projected via an LCD projector onto a transparent screen positioned over the subjects' head and viewed through a tilted mirror fixed to the head coil.

### 2.3 fMRI data acquisition

The anatomical and functional imaging was performed using identical 3 Tesla Siemens Trio scanners. High‐resolution T1 weighted scans were acquired using an MPRAGE sequence (Siemens). Functional imaging used an EPI sequence with a repetition time (TR) of 2,000 ms, echo time (TE) = 30 ms, flip angle = 90°, 220 mm field of view (FOV), 64 × 64 matrix. Functional slices were oriented 30° superior‐caudal to the plane through the anterior and posterior commissures in order to reduce signal dropout due to magnetic field in‐homogeneities (Deichmann, Gottfried, Hutton, & Turner, 2003). Each functional image was acquired in an interleaved way, comprising 34 4 mm axial slices for measurement of the blood oxygenation level‐dependent (BOLD) effect (Ogawa, Lee, Kay, & Tank, 1990), yielding 3.4 mm × 3.4 mm × 4.0 mm voxels.

### 2.4 fMRI data analysis

Image preprocessing and data analysis were performed using SPM8 (Wellcome Trust Centre for Neuroimaging). The preprocessing procedures have been described in our previous work (Kirk, Pagnoni, Hétu, & Montague, 2019). Briefly, preprocessing steps included motion correction, co‐registration, slice timing, normalization, and spatial smoothing. For the analysis, a general linear model (GLM) was applied to the fMRI time series where image onset was modeled as single impulse response functions including image duration and then convolved with the canonical hemodynamic response function (HRF).

A parametric regression analysis was used (Büchel, Holmes, Rees, & Friston, 1998; Phan et al., 2004) that allowed to model linear 1st order hemodynamic responses using orthogonalized polynomial expansions. This was performed for each of the two conditions (aversive and neutral images) using subject‐specific behavioral ratings for each image. Residual effects of head motion were corrected for by including the six estimated motion parameters for each subject as regressors of no interest. The procedures for first‐level and second‐level, random effects (RFX) analysis have been described in our previous work (Kirk et al., 2009). The statistical results given were based on a single‐voxel *t*‐statistics corresponding to *p* < .05 corrected for multiple comparisons using the false discovery rate statistic (FDR) with an extent threshold of >10 voxels. The co‐ordinates of all activations are reported in MNI space. Data were displayed using the xjView toolbox.

For the functional connectivity analysis, we implemented psychophysiological interaction analysis (PPI) (Friston et al., 1997). The PPI assess changes in functional connectivity between the seed region of the right amygdala and other brain regions whose activity covary with the amygdala. The PPI employed a regressor representing the deconvolved time series of neural activity within a 4‐mm sphere centered in the right amygdala (*x*,*y*,*z* = 20 –2 –16), which constituted the physiological variable. A second regressor representing the psychological variable, specifically the contrast [Aversive > Neutral]. Finally, a third regressor representing the cross product of the previous two (the PPI term). The model also included motion parameters as regressors of no interest. The PPI was carried out in each subject and entered into random effects analysis separately for each of the two groups.

## 3 RESULTS

### 3.1 Behavioral results

**FIGURE 1:**
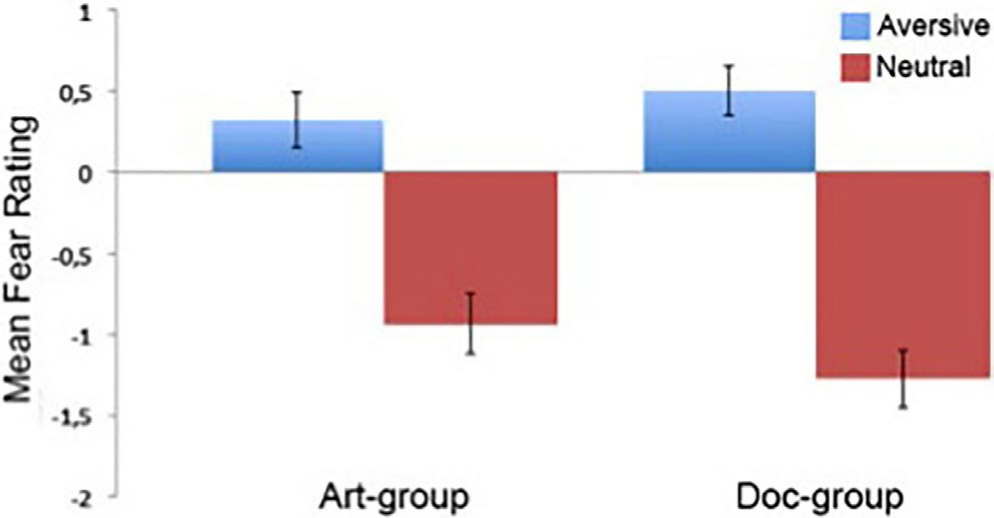
Mean behavioral responses. In the scanner, participants were presented with the IAPS images that were displayed for 5 s each during a passive scanning session. In a subsequent behavioral session, participants provided self‐paced fear ratings on a Likert‐type scale for each image. Participants were not informed about the behavioral task until after the scanning session. The mean rating and standard error (*SE*) for the art‐group aversive images were 0.31 (0.19) and neutral images −0.94 (0.20). The mean rating and *SE* for the doc‐group aversive images were 0.48 (0.14) and neutral images −1.31 (0.20). Statistical analysis showed no significant difference between the two groups

We were interested in seeing if there were differences in behavioral ratings for aversive and neutral images across the art and doc‐frame groups. Note that the behavioral ratings of the images were collected post hoc and thus not during the scanning run (see Methods). To further examine if there were any differences between the two groups a mixed ANOVA was used to inspect group (art‐frame, doc‐frame) by image type (aversive, neutral). There was no significant interaction between group and rating (*F* (1,37) = 2.01; *p* = .165). Similarly, there was not a significant main effect for group (*F* (1,37) = 0.26; *p* = .615). However, there was a substantial main effect for image type (*F* (1,37) = 65.63, *p* < .01, $\eta_{p}^{2}$ = 0.639) with both groups rating the aversive images more frightening than the neutral images (Figure 1).

### 3.2 fMRI results

**FIGURE 2:**
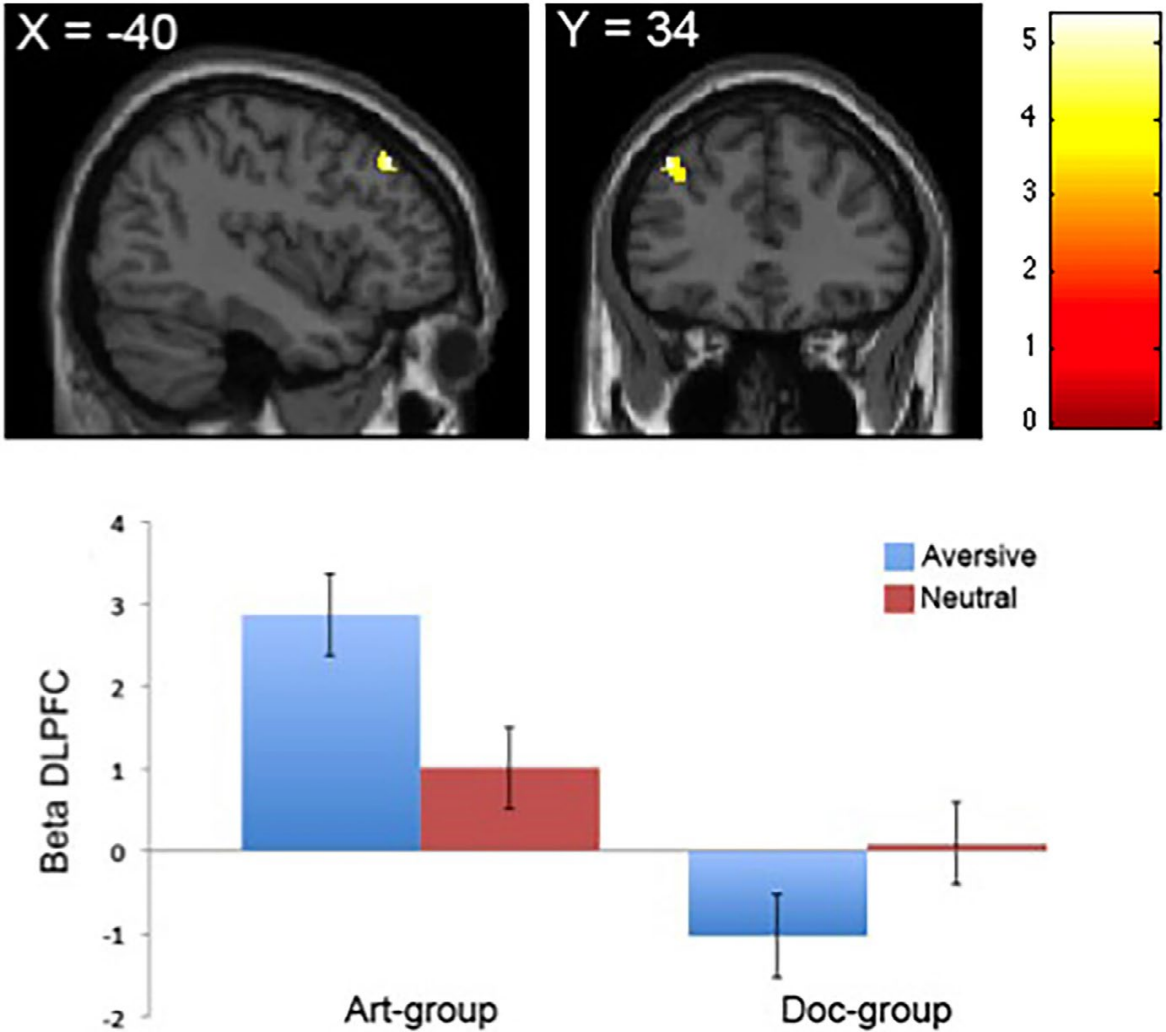
Neural activity in DLPFC in the main effect [art aversive > doc aversive]. Top: DLPFC display linear increase with fear responses for aversive images in the art‐frame group compared with the doc‐frame group. Bottom: Average beta values extracted for each group in the left DLPFC display better fit in terms of beta values for aversive images in the art‐frame group relative to the same images presented to the doc‐frame group. There are no differences between groups for neutral images. Error bars indicate *SE*

We aimed to identify neural regions that exhibited a modulation across the two groups when presented with aversive images. We used a parametric regression model that identifies areas of the brain that identifies areas of the brain whose activation amplitude scales linearly with subjective ratings. Note that we only report the linear effects (1st order polynomial expansions) and not the simple average effects (i.e., zero order polynomial expansions) in that the latter did not yield significant activations even when lowering the threshold (*p* < .001, uncorrected). When computing the contrast [art aversive > doc aversive], we found that the reactivity to fearful aversive images in the left DLPFC (*x*,*y*,*z* = −40 34 44; *p* < .05, FDR‐corrected; voxels = 35) exhibited a steeper slope related to the parametric analysis in the art‐frame group (Figure 2, top). We subsequently extracted β‐estimates from the DLPFC region and found that the aversive images in the art‐frame group drove the reactivity compared to the aversive images in the doc‐frame group (Figure 2, bottom). There were no differences across the groups with regard to the neutral images. These results suggest that the art frame does indeed recruit neural activity in regions associated with top‐down appraisal and cognitive control such as the DLPFC.

**FIGURE 3:**
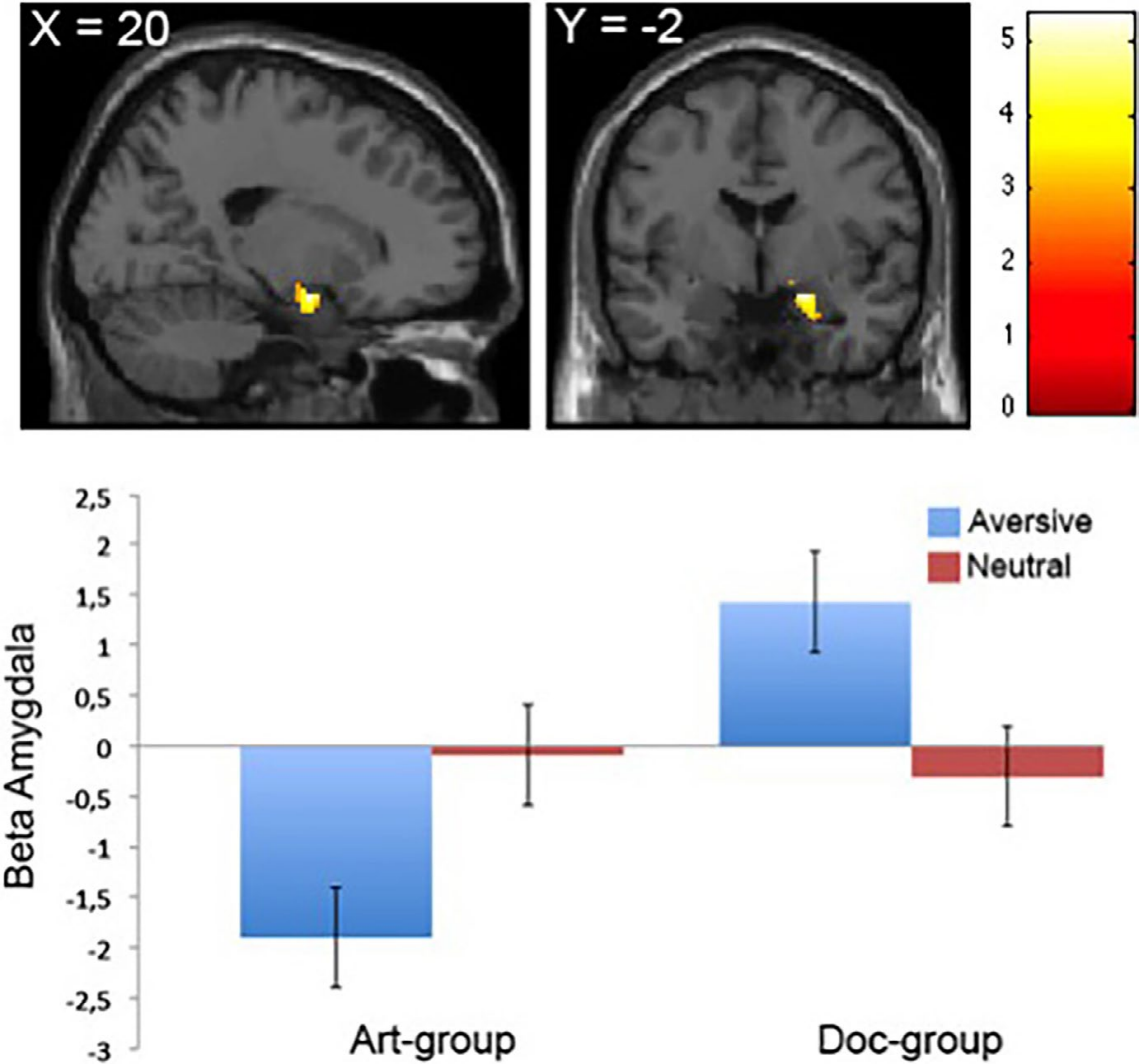
Neural activity in amygdala in the main effect [doc aversive > art aversive]. Top: Right amygdala display linear increase with fear responses for aversive images in the doc‐frame group compared with the art‐frame group. Bottom: Average beta values extracted for each group in the right amygdala display higher beta values for aversive images in the doc‐frame group relative to the same images presented to the art‐frame group. There are no differences between groups for neutral images. Error bars indicate *SE*

For the opposite contrast [doc aversive > art aversive], we found that the reactivity to fearful images in the right amygdala (*x*,*y*,*z* = 20 –2 –16; *p* < .05, FDR‐corrected; voxels = 43) exhibited a steeper slope related to the parametric analysis in the doc‐frame group (Figure 3, top). Furthermore, using average β‐estimates extracted from the right amygdala showed that the activation was driven by modulated reactivity in this region in the doc‐frame group (Figure 3, bottom).

**FIGURE 4:**
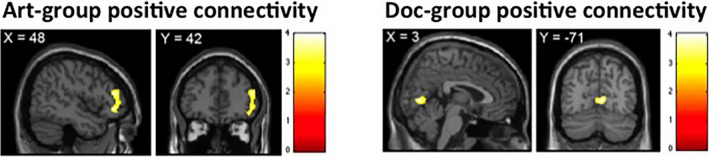
Group‐specific changes in effective connectivity for [aversive > neutral images]. Psychophysiological interaction analysis (PPI) displaying increased coupling between the right amygdala seed region and the right VLPFC in the art‐frame group. By contrast, the doc‐frame group displayed greater coupling between the amygdala seed region and the lingual gyrus in the visual cortex

We then assessed functional connectivity in the amygdala region of interest across the entire time series for the contrast [aversive > neutral images] separately for the two groups (Figure 4). An analysis of functional connectivity implemented as an psychophysiological interaction analysis revealed that the art‐frame group demonstrated a strong positive coupling with one region, in a whole brain analysis, namely the right ventrolateral prefrontal cortex (VLPFC) (*x*,*y*,*z* = 48 38 –6; *p* < .05, FDR‐corrected). This result suggests that in the art‐frame group, viewing aversive images compared to neutral ones is associated with a stronger amygdala‐VLPFC functional connectivity.

In contrast, the doc‐frame group showed a positive coupling with the visual cortex, specifically the lingual gyrus BA 18 (*x*,*y*,*z* = 3 –71 1; *p* < .05, FDR‐corrected). Such a strong correlation between a visual cortical region and the amygdala in processing aversive images may preclude a mode of disengagement of the aversive material depicted in the doc‐frame group.

Finally, we formally assessed the differences between the two PPI contrasts, albeit the did you yield significant voxels, even when lowering the threshold (*p* < .001, uncorrected).

## 4 DISCUSSION

The purpose of this study was to investigate how highly aversive pictures framed as art are processed compared to identical pictures framed as documentary by using fMRI. We hypothesized that the amygdala would reflect a steeper slope in the subjects belonging to the art‐frame group compared to the doc‐frame group. In line with this, we further hypothesized that amygdala activity would exhibit stronger coupling by VLPFC alongside DLPFC in the case of the art but not the doc‐frame group. In support of our hypotheses, the fMRI data showed that amygdala activity was down‐regulated while DLPFC was up‐regulated in subjects belonging to the art‐frame group. Conversely, for subjects belonging to the doc‐frame group amygdala activity exhibited a steeper slope in the parametric analysis. Moreover, framing aversive images as art was found to be associated with functional connectivity between amygdala and VLPFC, whereas the doc‐frame led to increased connectivity between amygdala and visual cortex. Taken together, these findings indicate that images framed as art lead to reactivity in regions associated with top‐down appraisal and cognitive control compared to images framed as documentary photographs. Consequently, the findings from this study suggest that framing highly aversive images as art, result in stronger VLFPC coupling of amygdalic activity. However, it should be noted that the formal comparison between the PPI contrast for the two groups did not yield significant voxels, which may suggest insufficient statistical power.

Although the fMRI data suggest that framing aversive visual stimuli as art recruit neural activity in regions associated with top‐down appraisal processes, this was not reflected in the behavioral rating task. Specifically, no significant difference was observed between behavioral ratings of aversive IAPS images across the art and doc‐frame groups. One possible explanation for this result is the well‐known fact that small sample sizes yield low statistical power (Button et al., 2013).

The fMRI data presented here are consistent with previous research which suggest that placing visual stimuli in an artistic context fosters top‐down appraisal processes aimed at inhibiting innate emotional responses. In extension, this result lends additional support to the empirical observation that appraising aversive stimuli as fictional rather than realistic reduces its emotional impact (Mocaiber et al., 2010, 2011). Accordingly, framing aversive images as art seems to provide the cognitive system with cues that fosters a specific style of perception allowing the observer to adopt a more distanced and reflective perspective of what is depicted. A possible explanation for this finding is that artistic stimuli, as opposed to real‐world stimuli, do not pose any risk for our survival and personal well‐being, due to its fictitious nature (Gerger et al., 2014). Hence, framing aversive stimuli as art eliminates the need for an adaptive emotional response such as fear, allowing for a more distanced and reflective perspective. In a similar vein, Scherer argues that artistic stimuli do not foster what he terms utilitarian emotions but rather what he refers to as aesthetic emotions (Scherer, 2004, 2005). Utilitarian emotions correspond to everyday emotions such as fear, joy anger, disgust, and sadness. These emotions are utilitarian in the sense that they facilitate adaptive response (e.g., fight, flight) to events that have important consequence for our survival and personal well‐being. On the contrary, aesthetic emotions do not have an adaptive function and are not shaped by the appraisal of the artwork's ability to satisfy physical needs or further current goals. Rather, aesthetic emotions, such as fascination, admiration, and rapture, are produced by the appreciation of the intrinsic qualities of the artwork. In line with Scherer's distinction between utilitarian and aesthetic emotions, it can thus be argued that activation of VLPFC‐DLPFC inhibits adaptive utilitarian emotions, allowing for a more distanced and reflective perspective in which aesthetic emotions can arise.

In agreement with previous neuroimaging studies, the fMRI data suggest that DLPFC is partially responsible for top‐down appraisal processes (Delgado et al., 2008; Kanske et al., 2011; Urry et al., 2006). Specifically, the fact that reactivity in DLPFC was found to be associated with amygdala reactivity strongly indicates that these brain regions are involved in top‐down appraisal processes aimed at regulating automatic emotional responses (Delgado et al., 2008; Kanske et al., 2011; Ochsner et al., 2002; Urry et al., 2006). Interestingly, the PPI analysis showed that subjects belonging to the art‐frame, but the not doc‐frame group demonstrated a significant positive coupling between VLPFC and amygdala. This is particularly interesting as it suggests that VLPFC plays an important role in top‐down appraisal processes by inhibiting automatic affective responses.

Although most appraisal theorists agree that context plays an important role in emotion regulation and appraisal processes, very few studies have investigated the effects of contextual framing (Aldao, 2013; Aldao & Tull, 2015; Gross, 2015). The results presented here are thus important: First because they indicate that contextual factors influence how people spontaneously process emotional stimuli; second because they show that people automatically engage in more effective emotion regulation strategies when highly aversive percepts are placed in an art context.

## 5 CONCLUSION

Building on the experimental framework put forward here future studies should strive to investigate how different contextual frames influence how people process highly emotional stimuli and indeed asking about subjects emotional experience (Kron, Goldstein, Lee, Gardhouse, & Anderson, 2013; Kron, Pilkiw, Banaei, Goldstein, & Anderson, 2015). Investigating this is an important next step in the study of emotions which would provide us with a more detailed understanding of the relationship between context and emotion processing and regulation.

## CONFLICT OF INTEREST

The authors declare no conflict of interest.

## AUTHOR CONTRIBUTION

UK and DF designed the study; UK and DF performed the research; UK and DF analyzed data; and UK, DF, and LL wrote the article.

## Data Availability

The results generated during the current study are available from the corresponding author on reasonable request.

## References

[brb31761-bib-0001] Aldao, A. (2013). The future of emotion regulation research. Perspectives on Psychological Science, 8, 155–172. 10.1177/1745691612459518 26172497

[brb31761-bib-0002] Aldao, A. , & Tull, M. T. (2015). Putting emotion regulation in context. Current Opinion in Psychology, 3, 100–107. 10.1016/j.copsyc.2015.03.022 PMC4386282 25859561

[brb31761-bib-0003] Barbas, H. (2000). Connections underlying the synthesis of cognition, memory, and emotion in primate prefrontal cortices. Brain Research Bulletin, 52, 319–330. 10.1016/S0361-9230(99)00245-2 10922509

[brb31761-bib-0004] Barbas, H. , & Pandya, D. N. (1989). Architecture and intrinsic connections of the prefrontal cortex in the rhesus monkey. The Journal of Comparative Neurology, 286, 353–375. 2768563 10.1002/cne.902860306

[brb31761-bib-0005] Büchel, C. , Holmes, A. P. , Rees, G. , & Friston, K. J. (1998). Characterizing stimulus–Response functions using nonlinear regressors in parametric fMRI experiments. NeuroImage, 8, 140–148. 10.1006/nimg.1998.0351 9740757

[brb31761-bib-0006] Button, K. S. , Ioannidis, J. , Mokrysz, C. , Nosek, B. , Flint, J. , Robinson, E. , & Munafò, M. (2013). Power failure: Why small sample size undermines the reliability of neuroscience. Nature Reviews Neuroscience, 14, 365–376. 23571845 10.1038/nrn3475

[brb31761-bib-0007] Cupchik, G. C. (2002). The evolution of psychical distance as an aesthetic concept. Culture & Psychology, 8, 155–187. 10.1177/1354067X02008002437

[brb31761-bib-0008] Cupchik, G. C. , Vartanian, O. , Crawley, A. , & Mikulis, D. J. (2009). Viewing artworks: Contributions of cognitive control and perceptual facilitation to aesthetic experience. Brain and Cognition, 70, 84–91. 19223099 10.1016/j.bandc.2009.01.003

[brb31761-bib-0009] Deichmann, R. , Gottfried, J. , Hutton, C. , & Turner, R. (2003). Optimized EPI for fMRI studies of the orbitofrontal cortex. NeuroImage, 19, 430–441. 10.1016/S1053-8119(03)00073-9 12814592

[brb31761-bib-0010] Delgado, M. R. , Nearing, K. I. , LeDoux, J. E. , & Phelps, E. A. (2008). Neural circuitry underlying the regulation of conditioned fear and its relation to extinction. Neuron, 59, 829–838. 10.1016/j.neuron.2008.06.029 18786365 PMC3061554

[brb31761-bib-0011] Friston, K. J. , Buechel, C. , Fink, G. R. , Morris, J. , Rolls, E. , & Dolan, R. J. (1997). Psychophysiological and modulatory interactions in neuroimaging. NeuroImage, 6, 218–229. 10.1006/nimg.1997.0291 9344826

[brb31761-bib-0012] Gerger, G. , Leder, H. , & Kremer, A. (2014). Context effects on emotional and aesthetic evaluations of artworks and IAPS pictures. Acta Psychologica, 151, 174–183. 24983515 10.1016/j.actpsy.2014.06.008

[brb31761-bib-0013] Goulas, A. , Uylings, H. B. M. , & Stiers, P. (2012). Unravelling the intrinsic functional organization of the human lateral frontal cortex: A parcellation scheme based on resting state fMRI. Journal of Neuroscience, 32, 10238–10252. 22836258 10.1523/JNEUROSCI.5852-11.2012 PMC6703746

[brb31761-bib-0014] Gross, J. J. (1998). Antecedent‐and response‐focused emotion regulation: Divergent consequences for experience, expression, and physiology. Journal of Personality and Social Psychology, 74, 224. 9457784 10.1037//0022-3514.74.1.224

[brb31761-bib-0015] Gross, J. J. (2015). Emotion regulation: Current status and future prospects. Psychological Inquiry, 26, 1–26. 10.1080/1047840X.2014.940781

[brb31761-bib-0016] Jacobsen, T. , Schubotz, R. I. , Höfel, L. , & Cramon, D. Y. V. (2006). Brain correlates of aesthetic judgment of beauty. NeuroImage, 29, 276–285. 10.1016/j.neuroimage.2005.07.010 16087351

[brb31761-bib-0017] Kanske, P. , Heissler, J. , Schönfelder, S. , Bongers, A. , & Wessa, M. (2011). How to regulate emotion? Neural networks for reappraisal and distraction. Cerebral Cortex, 21, 1379–1388. 10.1093/cercor/bhq216 21041200

[brb31761-bib-0018] Kirk, U. , Pagnoni, G. , Hétu, S. , & Montague, R. (2019). Short‐term mindfulness practice attenuates reward prediction errors signals in the brain. Scientific Reports, 9, 6964. 31061515 10.1038/s41598-019-43474-2 PMC6502850

[brb31761-bib-0019] Kirk, U. , Skov, M. , Hulme, O. , Christensen, M. S. , & Zeki, S. (2009). Modulation of aesthetic value by semantic context: An fMRI study. NeuroImage, 44, 1125–1132. 10.1016/j.neuroimage.2008.10.009 19010423

[brb31761-bib-0020] Kron, A. , Goldstein, A. , Lee, D. H. , Gardhouse, K. , & Anderson, A. K. (2013). How are you feeling? Revisiting the quantification of emotional qualia. Psychological Science, 24, 1503–1511. 10.1177/0956797613475456 23824581

[brb31761-bib-0021] Kron, A. , Pilkiw, M. , Banaei, J. , Goldstein, A. , & Anderson, A. K. (2015). Are valence and arousal separable in emotional experience? Emotion, 15, 35–44. 10.1037/a0038474 25664950

[brb31761-bib-0022] Lang, P. J. , Bradley, M. M. , & Cuthbert, B. N. (1997). International AffectivePicture System (IAPS): Technical Manual and Affective Ratings. Gainesville, FL: NationalInstitute of Mental Health Center for the Study of Emotion and Attention.

[brb31761-bib-0023] Lazarus, R. S. , & Alfert, E. (1964). Short‐circuiting of threat by experimentally altering cognitive appraisal. Journal of Abnormal and Social Psychology, 69, 195–205. 10.1037/h0044635 14213291

[brb31761-bib-0024] Leder, H. , Belke, B. , Oeberst, A. , & Augustin, D. (2004). A model of aesthetic appreciation and aesthetic judgments. British Journal of Psychology, 95, 489–508. 15527534 10.1348/0007126042369811

[brb31761-bib-0025] Mocaiber, I. , Perakakis, P. , Pereira, M. G. , Pinheiro, W. M. , Volchan, E. , de Oliveira, L. , & Vila, J. (2011). Stimulus appraisal modulates cardiac reactivity to briefly presented mutilation pictures. International Journal of Psychophysiology, 81, 299–304. 21820017 10.1016/j.ijpsycho.2011.07.014

[brb31761-bib-0026] Mocaiber, I. , Pereira, M. , Erthal, F. , Machado‐Pinheiro, W. , David, I. , Cagy, M. , … Oliveira, L. (2010). Fact or fiction? An event‐related potential study of implicit emotion regulation. Neuroscience Letters, 476, 84–88. 20385204 10.1016/j.neulet.2010.04.008

[brb31761-bib-0027] Nadal, M. , Munar, E. , Capo, M. A. , Rossello, J. , & Cela‐Conde, C. J. (2008). Towards a framework for the study of the neural correlates of aesthetic preference. Spatial Vision, 21, 379–396. 18534110 10.1163/156856808784532653

[brb31761-bib-0028] Ochsner, K. N. , Bunge, S. A. , Gross, J. J. , & Gabrieli, J. D. E. (2002). Rethinking feelings: An fMRI study of the cognitive regulation of emotion. Journal of Cognitive Neuroscience, 14, 1215–1229. 12495527 10.1162/089892902760807212

[brb31761-bib-0029] Ogawa, S. , Lee, T. M. , Kay, A. R. , & Tank, D. W. (1990). Brain magnetic resonance imaging with contrast dependent on blood oxygenation. Proceedings of the National Academy of Sciences of the United States of America, 87, 9868–9872. 2124706 10.1073/pnas.87.24.9868 PMC55275

[brb31761-bib-0030] Phan, K. L. , Taylor, S. F. , Welsh, R. C. , Ho, S. H. , Britton, J. C. , & Liberzon, I. (2004). Neural correlates of individual ratings of emotional salience: A trial‐related fMRI study. NeuroImage, 21, 768–780. 10.1016/j.neuroimage.2003.09.072 14980580

[brb31761-bib-0031] Scherer, K. R. (2004). Which emotions can be induced by music? What are the underlying mechanisms? And how can we measure them? Journal of New Music Research, 33, 239–251. 10.1080/0929821042000317822

[brb31761-bib-0032] Scherer, K. R. (2005). What are emotions? And how can they be measured? Social Science Information, 44, 695–729. 10.1177/0539018405058216

[brb31761-bib-0033] Speisman, J. C. , Lazarus, R. S. , Mordkoff, A. , & Davison, L. (1964). Experimental reduction of stress based on ego‐defense theory. Journal of Abnormal and Social Psychology, 68, 367–380. 10.1037/h0048936 14136776

[brb31761-bib-0034] Urry, H. L. , van Reekum, C. M. , Johnstone, T. , Kalin, N. H. , Thurow, M. E. , Schaefer, H. S. , … Davidson, R. J. (2006). Amygdala and ventromedial prefrontal cortex are inversely coupled during regulation of negative affect and predict the diurnal pattern of cortisol secretion among older adults. Journal of Neuroscience, 26, 4415–4425. 16624961 10.1523/JNEUROSCI.3215-05.2006 PMC6673990

[brb31761-bib-0035] Yeterian, E. H. , Pandya, D. N. , Tomaiuolo, F. , & Petrides, M. (2012). The cortical connectivity of the prefrontal cortex in the monkey brain. Cortex, 48, 58–81. 10.1016/j.cortex.2011.03.004 21481342 PMC3161133

